# Perfluorooctane Sulfonate Induces Dysfunction of Human Umbilical Vein Endothelial Cells via Ferroptosis Pathway

**DOI:** 10.3390/toxics10090503

**Published:** 2022-08-28

**Authors:** Jiajing Cui, Pingwei Wang, Shuqi Yan, Yujun Liang, Dongge Liu, Shuping Ren

**Affiliations:** Department of Occupational and Environmental Health, School of Public Health, Jilin University, Changchun 130021, China

**Keywords:** PFOS, HUVECs, ferroptosis, ROS, endothelial dysfunction

## Abstract

(1) Background: Perfluorooctane sulfonate (PFOS) is a persistent organic pollutant, and it is receiving increasing attention regarding its human health risks due to its extensive use. Endothelial dysfunction is a mark of cardiovascular disease, but the basic mechanism of PFOS-induced endothelial dysfunction is still not fully understood. Ferroptosis is a newly defined regulatory cell death driven by cellular metabolism and iron-dependent lipid peroxidation. Although ferroptosis has been shown to be involved in the pathogenesis of cardiovascular diseases, the involvement of ferroptosis in the pathogenesis of endothelial dysfunction caused by PFOS remains unclear. (2) Purpose: To explore the role of ferroptosis in the dysfunction of endothelial cells and underlying mechanisms. (3) Methods: Human umbilical vein endothelial cells (HUVECs) were exposed to PFOS or PFOS and Fer-1. The viability, morphology change under electronic microscope, lipid-reactive oxygen species (lipid-ROS), and production of nitric oxide (NO) were determined. The expression of glutathione peroxidase 4(GPX4), ferritin heavy chain protein 1 (FTH1), heme oxygenase 1 (HO-1) and Acyl-CoA synthetase long-chain family member 4 (ACSL4) were analyzed via Western blot analysis. (4) Results: PFOS was shown to cause a decrease in viability and morphological changes of mitochondria, and well as an increase in lipid droplets. The expression of GPX4, FTH1 and HO-1 was decreased, and that of ACSL4 was increased after exposure to PFOS. In addition to the above-mentioned ferroptosis-related manifestations, there was also a reduction in NO content. (5) Conclusions: PFOS induces ferroptosis by regulating the GPX4 and ACSL4 pathways, which leads to HUVEC dysfunction.

## 1. Introduction

Perfluorooctane sulfonate (PFOS), referred to as PFOS, is an artificial synthetic chemical. As a surfactant, it is widely used in commerce and industry, including in wetting agents, lubricants, preservatives, pesticides, and foam extinguishers [1]. As a persistent organic pollutant, PFOS possesses the characteristics of high toxicity, persistence, bioaccumulation and long-distance migration [2]. There is limited information on the toxicokinetics of PFOS in humans, but it is generally believed that PFOS in human serum rather than in plasma is the main indicator of PFOS load in vivo [3]. In recent years, biomonitoring studies have detected significant increases in PFOS levels in the serum of people around the world. For example, the average serum concentration of EU citizens is 7.7 ng/mL in adults and 3.2 ng/mL in children [4]. The authors of another study analyzed 100 human plasma samples collected in the German Environmental Sample Bank (ESB) from 2009 to 2019, and the PFOS levels detected in each sample were at concentrations of 1.21–14.1 ng/mL [5]. Developed countries typically detect higher values than developing countries, and cities with more developed industries and dense populations detect higher values [3].

Once PFOS enters the body, it can hardly be metabolized [6]. Some studies have suggested that dietary contact is the main contact pathway for adults, and other contact sources include drinking water, dust and household consumer goods treated with PFOS [7,8,9]. It is estimated that the average serum half-life of PFOS is 5.4 years [10]. This long half-life indicates that these compounds are bioaccumulative, which may lead to higher body burdens and more adverse health effects. PFOS has been detected in maternal and fetal umbilical cord blood samples [11]. It can penetrate the placental barrier, and its concentration in umbilical cord blood was shown to be from 1.5 to 3.5 times lower than that in maternal blood [12]. Studies have explored the potential role of PFOS in various vascular inflammatory diseases and endothelial dysfunction, but the exact mechanism underlying these effects remains unclear [13].

Ferroptosis is a new type of programmed cell death that is iron-dependent and different from apoptosis, cell necrosis, and autophagy. It catalyzes the lipid peroxidation of highly expressed unsaturated fatty acids on the cell membrane under the action of Fe^2+^ or lipoxygenase, thereby inducing cell death [14,15]. Iron metabolism and lipid peroxidation signal transduction are considered to be the key media for iron death. Excessive iron is stored in ferritin, which is a complex of iron storage proteins, including iron light chain protein (FTL) and ferritin heavy chain protein 1 (FTH1) [15]. FTH has iron oxidase activity, which can catalyze the transformation of ferrous form (Fe^2+^) to ferrous form (Fe^3+^),and promote the safe entry of iron into ferritin, thereby reducing the free iron level [16]. Iron can also be released by heme oxygenase 1 (HO-1) during heme degradation. HO-1 is a stress-induced enzyme encoded by the Hmox1 gene [17], and its up-regulation can protect cells from iron death [18].

In morphology, iron death is mainly characterized by the obvious atrophy of mitochondria, increased membrane density, and decreased or disappeared mitochondrial crista, which make its different from other types of cell death [14]. Biochemically, Fe^2+^ oxidizes lipids in a Fenton-like manner, resulting in a large number of lipid-reactive oxygen species (lipid-ROS) [19]. When the antioxidant defense system cannot achieve a balance of ROS, oxidative stress occurs [14,20]. Many iron cell growth regulators, such as Acyl-CoA synthetase long-chain family member 4(ACSL4) and glutathione peroxidase 4(GPX4), have been shown to regulate ferroptosis [15,21,22]. 

Endothelial dysfunction in cardiovascular complications is mainly manifested as enhanced oxidative stress, the reduced release of nitric oxide (NO), the increased production of inflammatory factors, impaired endothelial repair, and abnormal angiogenesis [23]. In addition, enhanced oxidative stress and the inactivation of NO by ROS are the main features of endothelial dysfunction [24]. PFOS can destroy the balance of the antioxidant system, leading to oxidative damage and even apoptosis, which provides evidence that PFOS may induce ferroptosis [6]. Although PFOS exposure has been confirmed to play an important role in vascular inflammatory disorder and endothelial dysfunction [13], ferroptosis related to oxidative stress has not been fully linked to the pathology of vascular diseases, which is of great significance in the clarification of the molecular mechanism for controlling endothelial cell death and improving the treatment of vascular diseases. Therefore, we aimed to explore the potential role and underlying ferroptosis-related mechanisms of PFOS in the dysfunction of human umbilical vein endothelial cells (HUVECs).

## 2. Materials and Methods

### 2.1. Cell Culture

HUVECs (purchased from Shanghai Institute of Biochemistry and Cell Biology, Chinese Academy of Sciences) were cultured in an RPMI Medium 1640 (GIBCO- Thermo Fisher, Waltham, MA, USA) containing 10% fetal bovine serum (FBS Biological industry, Kibbutz Beit-Haemek, Israel) and 1% penicillin and streptomycin (Solarbio, Beijing, China).

Cells were cultured at 37 °C and 5% CO_2_, and when the confluence of the cells was 70–80%, the cells were treated as follows: The cells in the control group were not treated, the cells in the DMSO group were treated with 0.1% DMSO (Sigma, St. Louis, MO, USA) for 12 h as a solvent control, the cells in the PFOS group were treated with PFOS (180 μM, Weng Jiang Reagent, China) for 12 h, and the cells in the Fer-1 and PFOS group were treated with Fer-1 inhibitor (1 μM, Abcam, Boston, MA, USA) for 2 h and then treated with PFOS (180 μM, Weng Jiang Reagent, Guangdong, China) for 12 h. PFOS and Fer-1 were dissolved in DMSO. The concentration and incubation time of PFOS were determined by the results of a cell viability assay. The concentration of Fer-1 was selected based on a previous study [25].

### 2.2. Cell Viability Assay

Cells were seeded into a 96-well plate with 10,000 cells in 100 μL of medium per well. After exposure to 100, 150, 200, 250, 300, 400 or 500 μM PFOS or an equal volume of DMSO for 12, 24, or 48 h, the Cell Counting Kit-8 (CCK-8) assay was performed to determine cell viability. The cells were incubated with 10 μL of CCK-8 (APE*BIO, Houston, TX, USA) for 2 h at 37 °C in the dark. Absorbance was measured at 450 nm using a microplate spectrophotometer. Cell viability was calculated as the ratio of the mean OD value among the cells treated differently in each group. IC50 values were calculated via dose–response curve fitting using GraphPad Prism v8.0.2 software (San Diego, CA, USA, 2018).

### 2.3. Transmission Electron Microscope

The four above-mentioned groups of cells were cultured in 60 mm plates, collected in a phosphate-buffered saline (PBS) solution, and fixed with 2% (*v*/*v*) paraformaldehyde (PFA, Beyotime, Shanghai, China) containing 2.5% (*w*/*v*) glutaraldehyde (Sigma, St. Louis, MO, USA) buffered in Hank’s modified salt solution (HMSS, Procell, Wuhan, China) at 4 °C for 4 h. The cells were further fixed in a 1% (*w*/*v*) OsO4 solution buffered by 0.1 M cacodylate (pH 7.2) at 4 °C for 2 h. Then, the cells were scraped off the plastic and dehydrated with dinethanol. Dehydration was conducted in propylene oxide. The specimens were embedded in an EPON medium and cut into 60–70 nm sections. The samples were analyzed and recorded with a JEOL 1200 electron microscope (JEOL Ltd., Tokyo, Japan). 

### 2.4. Lipid-ROS Assay by Flow Cytometry

The intracellular lipid-ROS generation was measured using C11 BODIPY 581/591 (Thermo Fisher, Waltham, MA, USA). The collected cells from the four above-mentioned groups were resuspended with RPMI-1640 containing C11 BODIPY (10 μM) and incubated at 37 °C for 1 H. The cells were washed with a serum-free cell culture medium three times to remove the C11 BODIPY that did not enter the cells. Then, the cells were analyzed using flow cytometry. Intracellular fluorescence signal intensity reflecting intracellular lipid-ROS level was measured with flow cytometry (FACS; CantoIIflow cytometer, Becton Dickinson, Franklin Lakes, NJ, USA). The corresponding X-axis values represent the relative fluorescence intensities under same cell counts.

### 2.5. Western Blot Analysis

The cells were washed twice with PBS, lysed in a 100 μL protein lysis buffer (RIPA: PMSF = 100:1, Solarbio, Beijing, China), and centrifuged at 12,000 rpm at 4 °C for 15 min, and then the supernatant was frozen at −80 °C. The protein concentration was determined using BCA (Beyotime, Shanghai, China), in which equal amounts of protein (60 μg) were subjected to SDS-PAGE on a 10% or 12% gel. The separated proteins were electrophoretically transferred to PVDF membranes and 5% degreased milk was used asblocking agent for 2 h at room temperature and incubated with primary antibodies overnight at 4 °C. Subsequently, the membranes were incubated with horseradish peroxidase-labeled antibodies for 1 h at room temperature. The signals were detected using an enhanced chemiluminescence reagent (Bio-Rad 170-5060, Hercules, CA, USA). The primary antibodies included mouse anti-GADPH (1:1000, Abcam, Boston, MA, USA), rabbit anti-FTH1 (1:2000, Abcam, Boston, MA, USA), rabbit anti-HO-1 (1:2000, CST, Danvers, MA, USA), rabbit anti-ACSL4 (1:10,000, Abcam, Boston, MA, USA), and rabbit anti-GPX4 (1:2000, CST, Danvers, MA, USA). 

### 2.6. Nitric Oxide (NO) Content Determination

The NO contents in the supernatants of the cell culture media were determined using a Micro NO Content Assay Kit (Solarbio, Beijing, China). Taking the concentration of each standard solution as the x axis (x, μmol/mL) and the corresponding ΔA standard as the y axis (y, ΔA standard), we drew a standard curve to obtain the standard equation y = kx + b; then, the ΔA determination was brought into the equation to obtain x (μmol/mL). The calculation of NO contents was performed as follows: the number of sample cells (μmol/104 cells) = x × V samples ÷ (V samples × number of cells ÷ total V samples) = x ÷ number of cells.

### 2.7. Statistical Analysis

All tests regarding data acquisition were conducted thrice, and all relevant data are displayed as mean ± standard deviation. The determination of the significant differences among multiple groups in the respective treatments were performed using the one-way analysis of variance (ANOVA) using GraphPad Prism version 8.0.2 (GraphPad Software, San Diego, CA, USA, 2018) and IBM SPSS 24.0 (IBM Corp., Armonk, NY, USA, 2016). The significance of the difference between various groups of data is shown as a *p*-value, and the *p*-values are shown in figures as follows: No significant difference (NS), *p* > 0.05; *, 0.05 > *p* > 0.01; **, *p* < 0.01; ***, *p* < 0.001.

## 3. Results

### 3.1. PFOS Can Inhibit the Multiplication and Viability of HUVECs In Vitro

**Figure 1:**
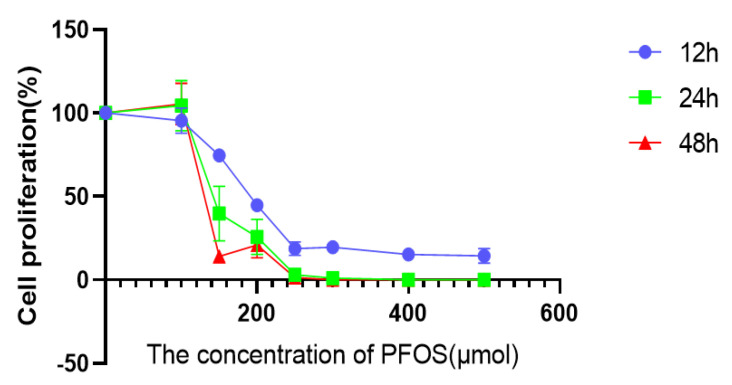
Effects of PFOS on the viability of HUVECs. Cell viability after HUVECs were exposed to PFOS.HUVECs were exposed to PFOS at the concentration of 0 µM, 100 µM, 150 µM, 200 µM, 250 µM, 300µM, 400µM, 500µM for 12 h and CCK−8 was used to detect the cell viability; the results were from three independent experiments.

To investigate the effects of PFOS on the multiplication and viability of HUVECs, a CCK-8 experiment was performed. The results showed that the viability of HUVECs was significantly reduced by the treatment of PFOS for 12 h. Moreover, the higher the PFOS concentration, the lower of the viability of HUVECs. Cell viability was also measured at 24 h and 48 h after PFOS treatment. It was found that prolonged drug treatment time increased the negative effect of PFOS on the cell viability of HUVECs, and it could be concluded that this effect was proportional to drug concentration and treatment time (Figure 1). However, the data showed that when the drug concentration was less than 100 μM, no matter how long the action time was, there was little effect on cell viability; when the drug concentration was greater than 220 μM, the cell viability reached its lowest level and there was no longer a linear trend between drug concentration and cell viability. Taken together, these data suggest that PFOS has inhibitory effects on the proliferation and in vitro viability of HUVECs. When the action time was 12 h, the IC50 value of PFOS was 180 μM, which was used as the drug action condition for subsequent experiments.

### 3.2. Ferroptosis of HUVECs Observed under Transmission Electron Microscope

**Figure 2:**
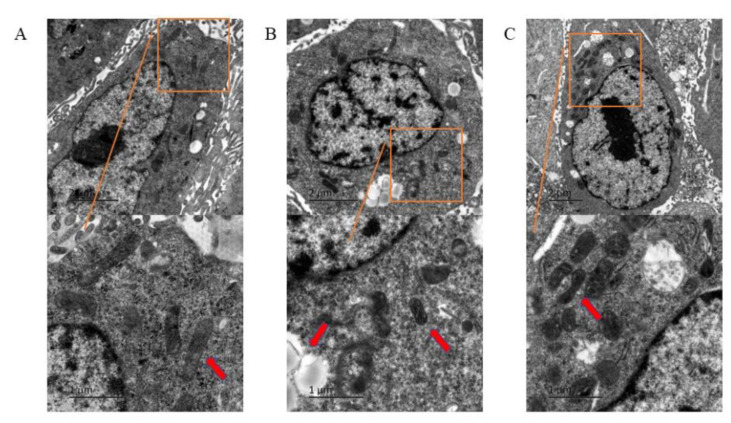
The morphological changes of HUVECs under electron microscope. HUVECs were treated with DMSO, PFOS (180 μM), or Fer-1 (1 μM) and PFOS (180 μM). After treatment, the cells were collected and fixed in Hank’s modified salt solution and analyzed with a JEOL 1200 electron microscope. (**A**) Control group; (**B**) PFOS group; (**C**) PFOS+Fer-1 group.

In the PFOS group (180 μM), a larger number of lipid droplets were observed in the cells compared with the control group (Figure 2A). Some of the cells had smaller and atrophic mitochondria, decreased mitochondrial cristae, and increased mitochondrial membrane density (Figure 2B). In the PFOS and Fer-1 group, lipid droplets were reduced, mitochondrial volume was larger, and atrophy was lower (Figure 2C). The above-mentioned microstructure changes showed that PFOS treatment caused ferroptosis in HUVECs.

### 3.3. PFOS Can Increase the Production of ROS

**Figure 3:**
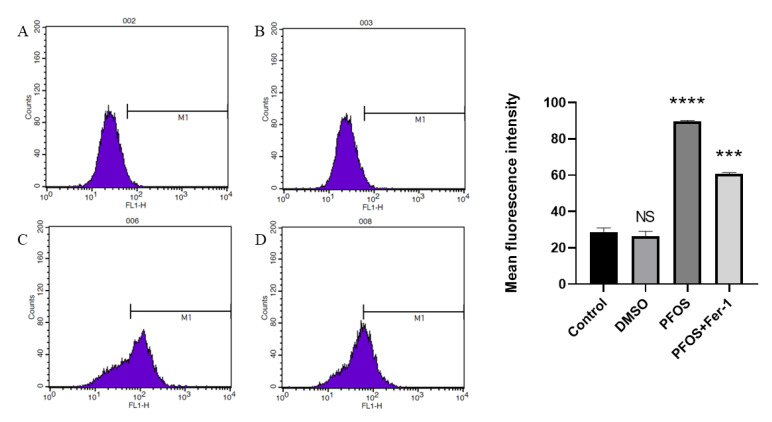
Detection of ROS production by flow cytometry. HUVECs were treated with DMSO, PFOS (180 μM), or Fer-1 (1 μM) and PFOS (180 μM). Then, the cells were collected and incubated with RPMI-1640 containing C11 BODIPY (10 μM) for 1 h. After incubation, the cells were washed with a serum-free cell culture medium and analyzed with flow cytometry. (**A**) Control group; (**B**) DMSO group; (**C**) PFOS group; (D) PFOS+ Fer-1 group. Each group was compared with the control group. NS, *no significant difference*; ***, *p* < 0.001; ****, *p* < 0.0001.

Compared with the cells from the control group (Figure 3A,B), the cells exposed to PFOS produced significantly higher levels of lipid-ROS, and the cells exposed to PFOS and Fer-1 (Figure 3D) produced lower level of lipid-ROS compared with those exposed to PFOS (Figure 3C). This further confirmed that PFOS could induce ferroptosis in HUVECs.

### 3.4. PFOS Can Induce Ferroptosis via Ferroptosis-Related Pathway

**Figure 4:**
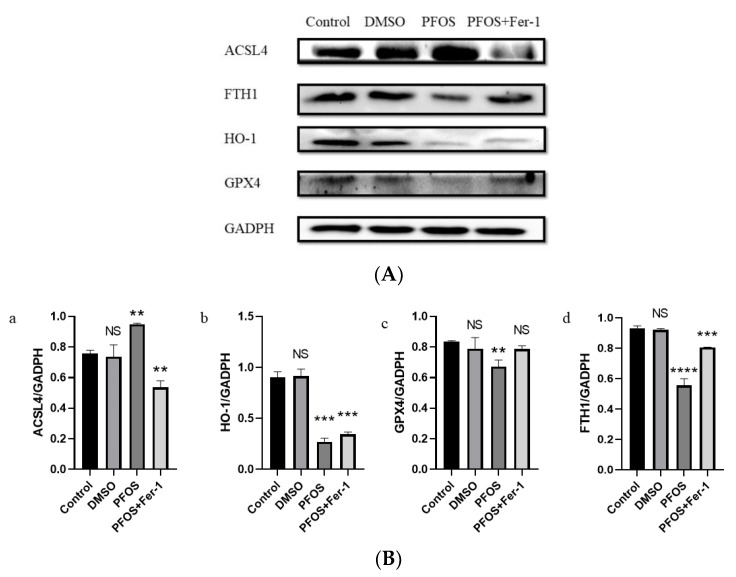
Effects of PFOS on the expression of ferroptosis-related proteins. HUVECs were treated with DMSO, PFOS (180 μM), or Fer-1 (1 μM) and PFOS (180 μM). Then, the cells were collected and the protein was extracted. The separate proteins were detected with enhanced chemiluminescence reagent. (**A**) Western blot analysis of the expression of ferroptosis-related proteins. GAPDH was recruited as a loading control. (**B**) Quantification analysis of the expression of ferroptosis-related proteins. Data are presented as the means ± SD (*n* = 3). Each group was compared with the control group. NS, *no significant difference*; **, *p* < 0.01; ***, *p* < 0.001; ****, *p* < 0.0001.

Western blot analysis demonstrated that PFOS could increase the expression of ACSL4 and decrease the expression of GPX4, HO-1, and FTH1. With the pre-treatment of Fer-1, the expression of ACSL4 was decreased (Figure 4(B-a) and the expression of GPX4 and FTH1 was increased (Figure 4(B-c,B-d)). There was a significant difference in the expression of HO-1 between the PFOS group and the PFOS and Fer-1 group (Figure 4(B-c)).

### 3.5. PFOS Can Decrease the Production of NO 

**Figure 5:**
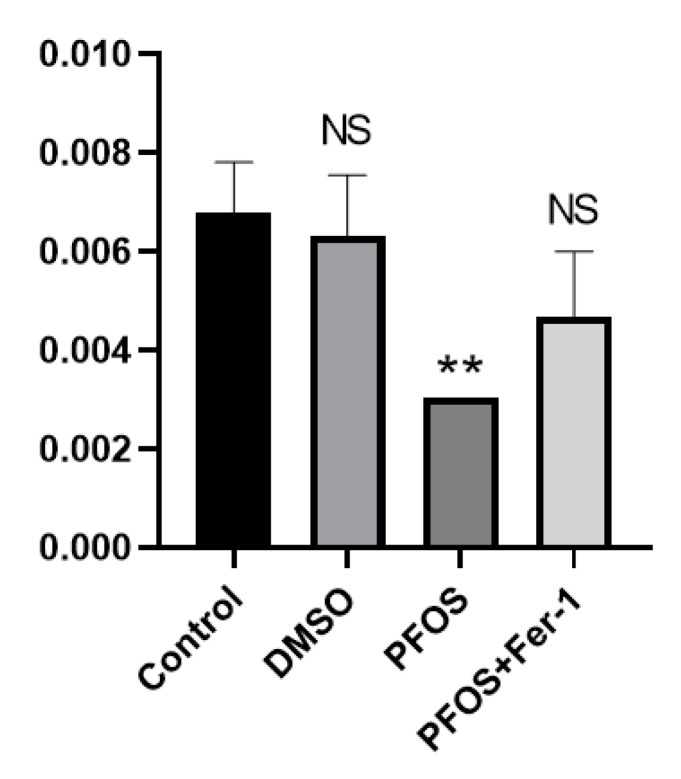
Effects of PFOS on the production of NO. HUVECs were treated with DMSO, PFOS (180 μM), or Fer-1 (1 μM) and PFOS (180 μM). The supernatants of the cell culture media were collected and the NO content was determined using a Micro NO Content Assay Kit. Each group was compared with the control group. NS, *no significant difference*; **, *p* < 0.01.

The production of NO in the cells exposed to PFOS was significantly decreased, and there was no significant difference in the production of NO between the cells exposed to PFOS and Fer-1 and the cells from the normal group (Figure 5). It was shown that Fer-1 could alleviate the symptoms of NO reduction caused by PFOS and improve the state of NO inactivation, which further indicates that PFOS can cause cell damage through ferroptosis.

## 4. Discussion

Despite considerable progress in diagnosis, prognosis, and treatment, cardiovascular disease (CVD) is still the main cause of morbidity and mortality worldwide [26]. Genetic factors cannot fully explain the sharp rise in the incidence of cardiovascular diseases in recent years, among which the PFAS family, especially PFOS, plays an important role in the development of CVD [27]. Some studies have evaluated the health risks of PFOS to human beings, and the widely studied toxic mechanisms of PFOS are oxidative stress and physiological process damage based on fatty acid similarity [28]. Ferroptosis induced by oxidative stress plays a key role in vascular heart disease [29], and whether this effect exists in endothelial cells has not been studied. PFOS can produce direct cardiovascular toxicity to humans [28]; although this conclusion is mostly based on animal experiments, the findings of our experiments also verify it. 

Ferroptosis is a newly defined iron-dependent form in the process of regulatory cell death (RCD) that is induced by excessive lipid peroxidation [14,30,31]. Increased lipid levels may also induce endothelial cell dysfunction and death by increasing oxidative stress [32]. The results of the presented experiments showed that PFOS induced ferroptosis in HUVECs, resulting in increased lipid-ROS levels and decreased cell proliferation and in vitro viability, which was proven by the protective effect of Fer-1. Many studies have shown that GPX4 is the main target of ferroptosis [33], and inhibiting FTH1 can increase the incidence of ferroptosis [34]. In this study, the expression of ferroptosis biomarker ACSL4 increased, the expression of GPX4 and FTH1 decreased, and the expression of HO-1 decreased in the PFOS group, results consistent with the study of Yeru Chen on the relationship between ferroptosis and ulcerative colitis [35]. In addition, the activation of ACSL4 enhances ferroptosis in HUVECs. As Feng-JunXiao et al. proposed, the imbalance of ACSL4/A20 axis-mediated endothelial ferroptosis is closely related to the pathogenesis of ischemic diseases [36]. Some studies have shown that ACSL4 is responsible for shaping the cell lipid group by acting as an important node to determine the sensitivity and resistance to ferroptosis [22], which is the possible mechanism of ferroptosis in endothelial cells. 

Previous studies have confirmed that endothelial dysfunction is one of the most important factors in cardiovascular diseases, and it is considered to be the main change involved in the pathogenesis of macrovascular diseases [37]. Endothelial dysfunction is the imbalance of vasoconstrictors (such as prostaglandins, angiotensin II, and endothelin-1) and vasodilators (such as prostaglandins, NO, and endothelial-derived hyperpolarizing factors that can lead to vasoconstriction and vascular inflammation) [38]. In endothelial cells, nitric oxide (NO) is the main reason for maintaining vascular homeostasis. The decreased bioavailability of NO is due to decreased NO production and/or increased NO degradation by superoxide anions, which marks the beginning of endothelial dysfunction. Specifically, superoxide anions react with NO, which leads to the formation of peroxynitrite. In turn, peroxynitrite promotes protein nitrification and leads to endothelial cell dysfunction and death [32]. In this study, although NO content in the Fer-1 group was less than that in the normal group, it was significantly less than that in the PFOS group, which indicated that ferroptosis played an indispensable role in reducing NO. 

As an endocrine disruptor, PFOS has been found to be associated with the development of more diseases besides cardiovascular disease, such as endometriosis and polycystic ovary syndrome (PCOS)-related changes, especially pregnancy complications, endometrial abnormalities, and infertility [39,40,41,42]. In addition to epidemiological studies, the development and utilization of exposure biomarkers and effector biomarkers to elucidate toxicokinetic and toxicokinetic pathways requires support from toxicological studies. This study links PFOS to ferroptosis and cardiovascular disease, providing evidence that ferroptosis plays an important role in endometriosis and PCOS.

In summary, the findings of this study showed that PFOS induced the expression of oxidative stress and ferroptosis-related pathways, resulting in increased ROS levels and decreased NO content and ultimately leading to endothelial ferroptosis and endothelial dysfunction. From the perspective of ferroptosis, we have described the new mechanism of endothelial vascular injury, and further animal experiments are needed to prove our proposal.

## 5. Conclusions

In PFOS-induced vascular diseases studied here, ferroptosis-related pathways were activated, GPX4 was down-regulated, and ACSL4 was up-regulated, resulting in increased lipid reactive oxygen species levels, triggering iron death in endothelial cells and eventually leading to endothelial dysfunction. In summary, our study has shown for the first time that PFOS is associated with ferroptosis and endothelial dysfunction and that PFOS plays a vital role in the ferroptosis and endothelial dysfunction of endothelial cells. These facts support the hypothesis that the inhibition of ferroptosis can attenuate endothelial dysfunction in cardiovascular diseases.

## Data Availability

Not applicable.
